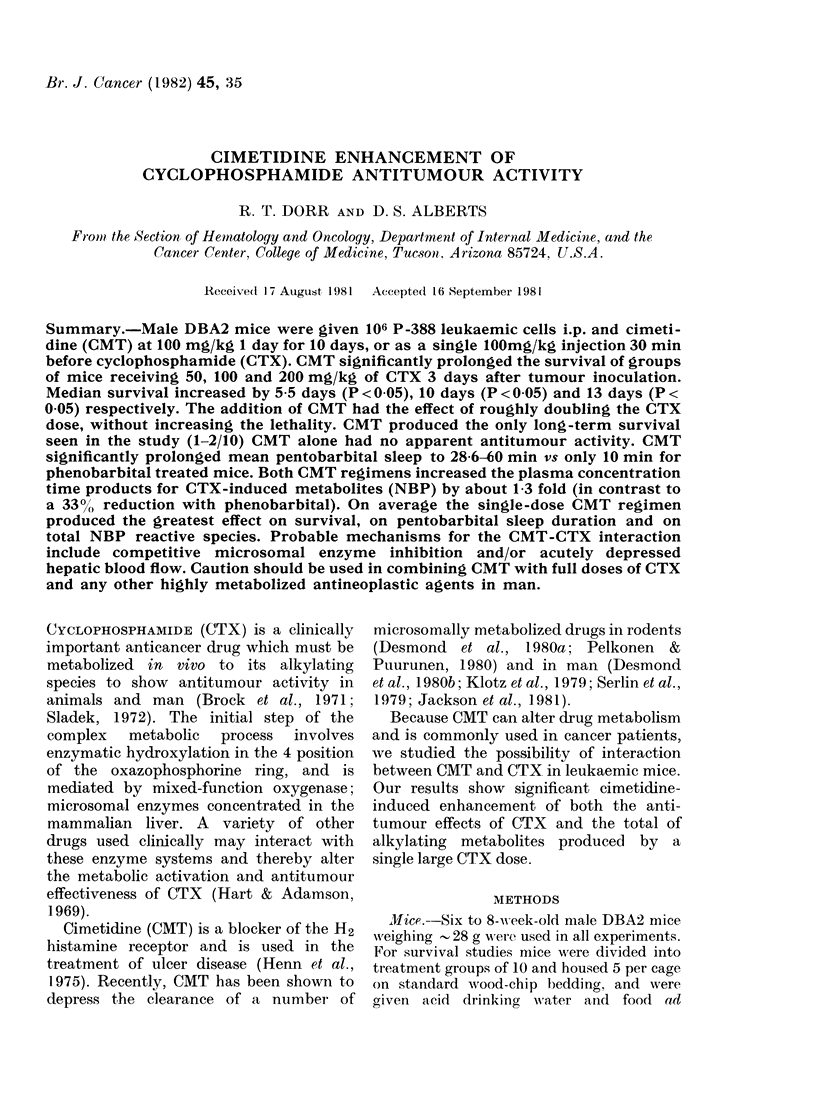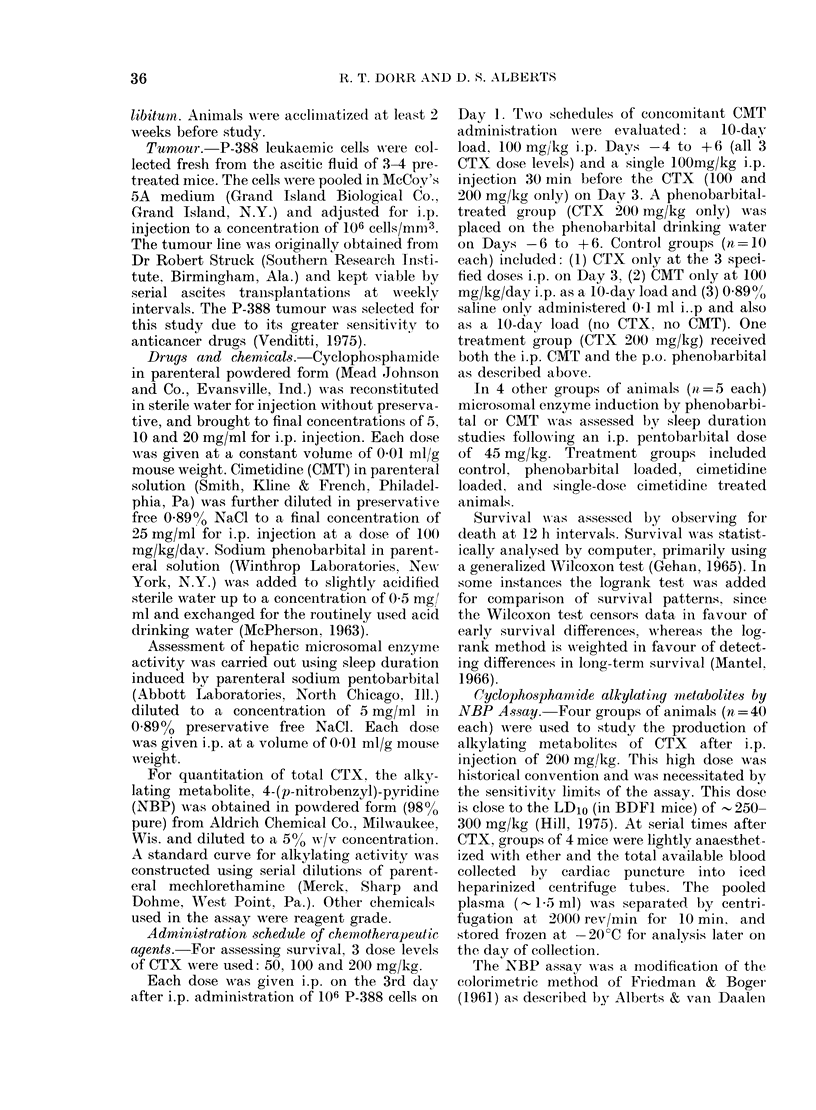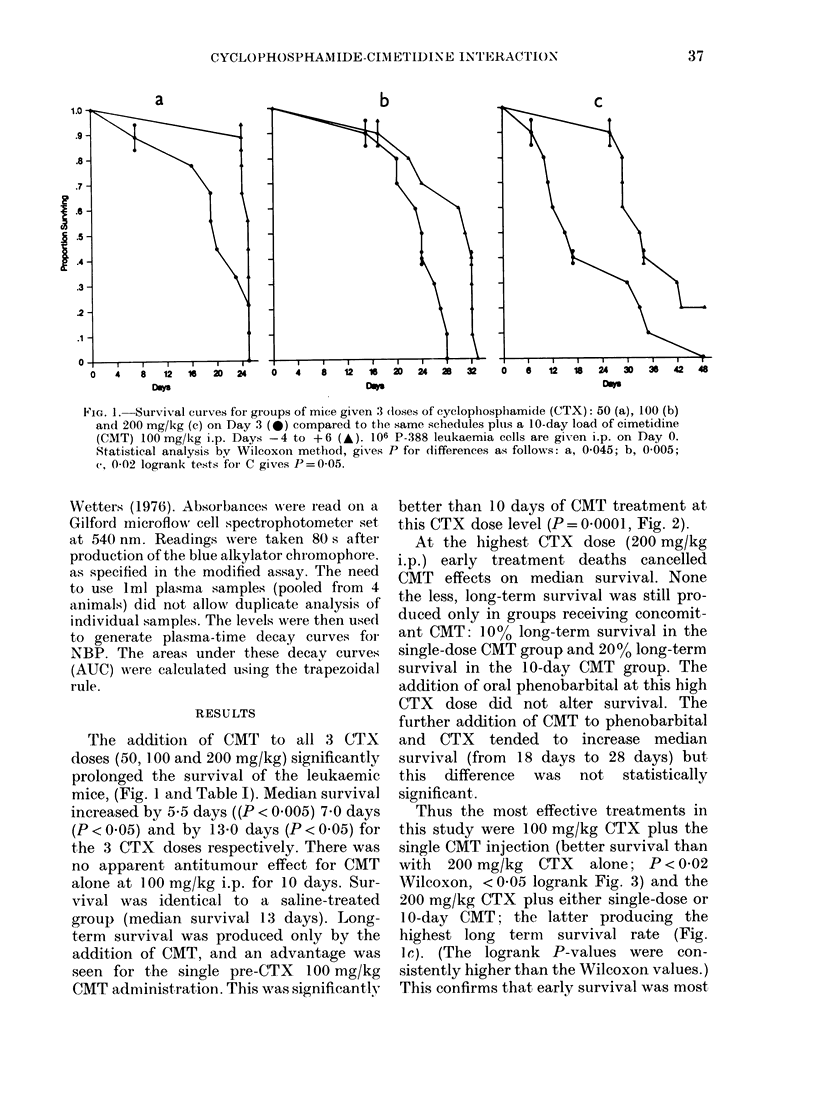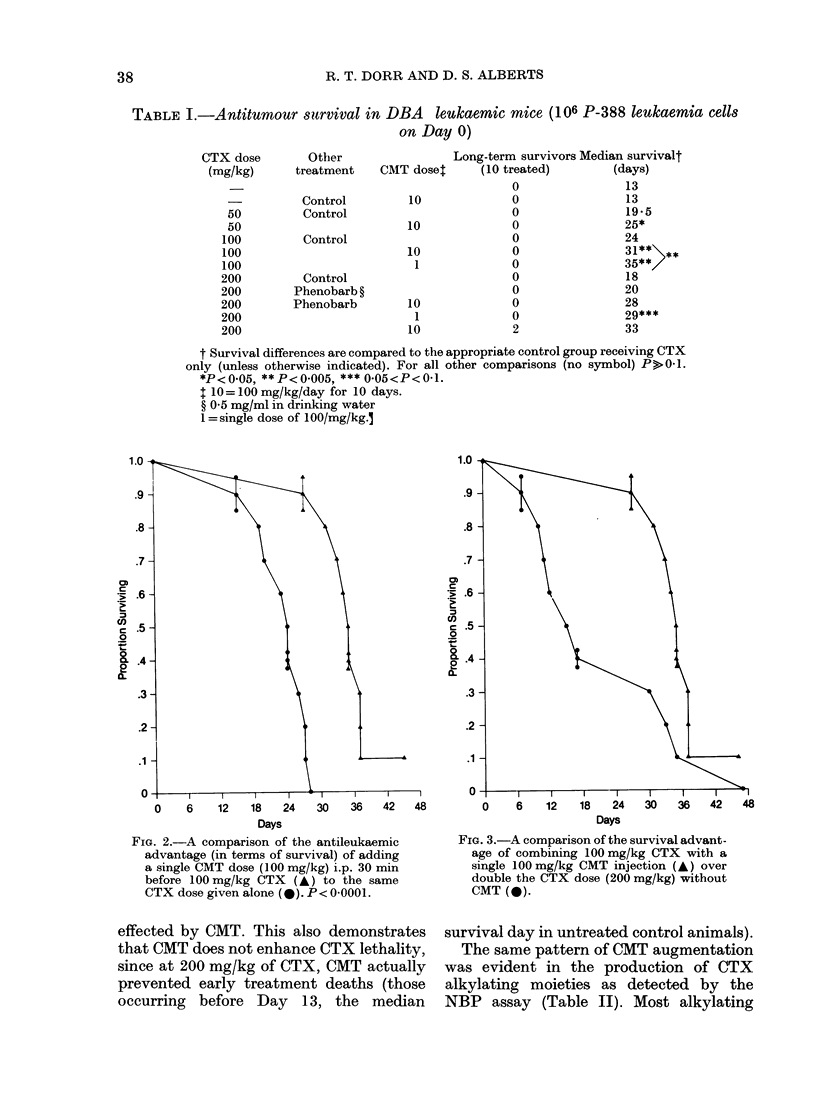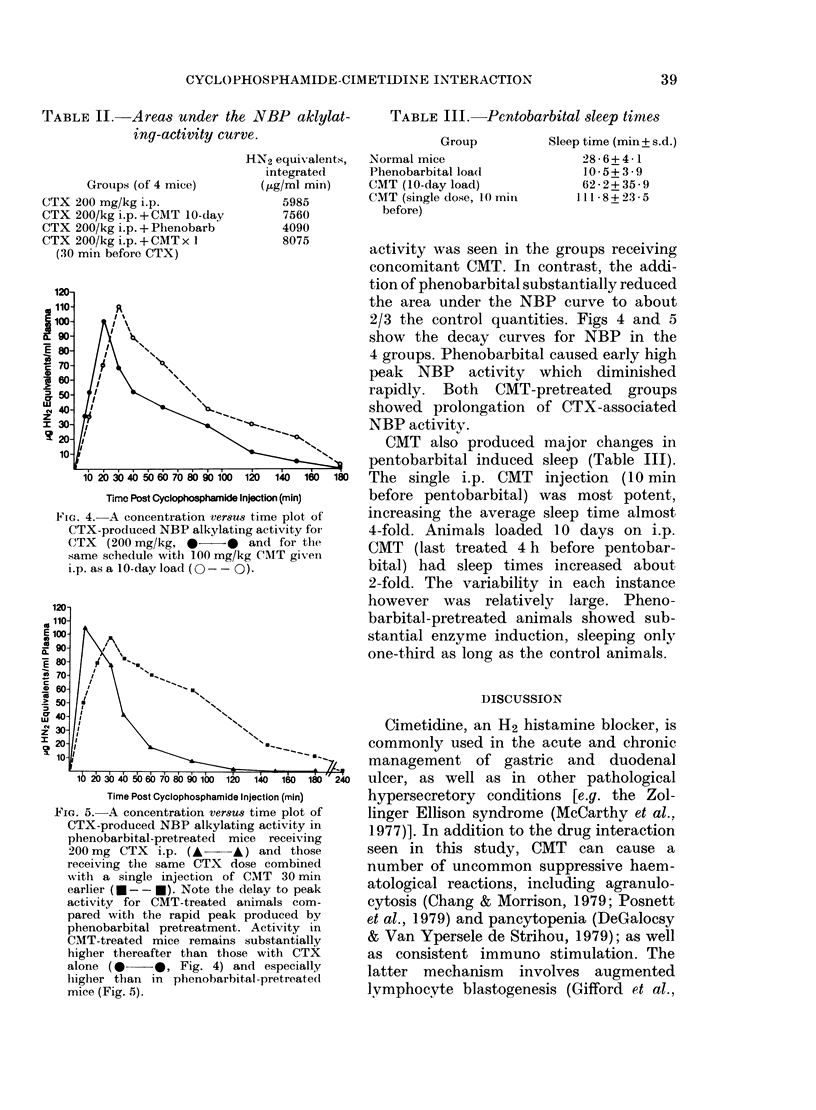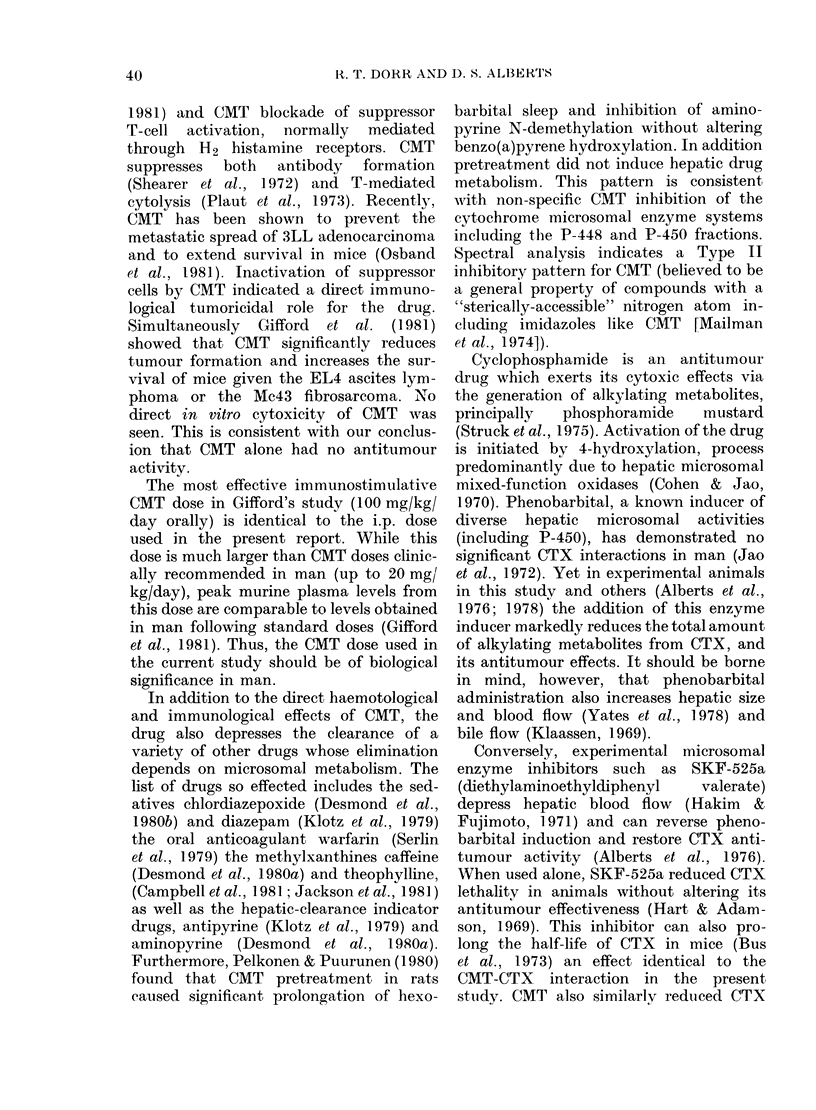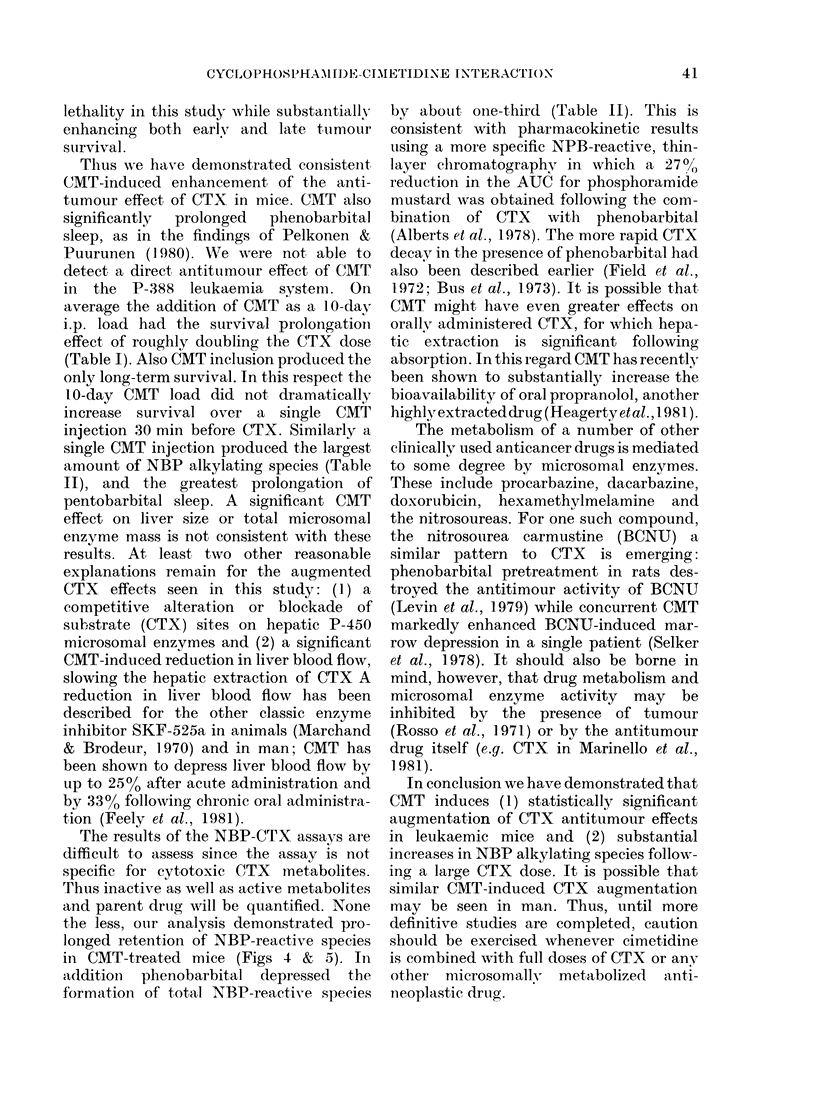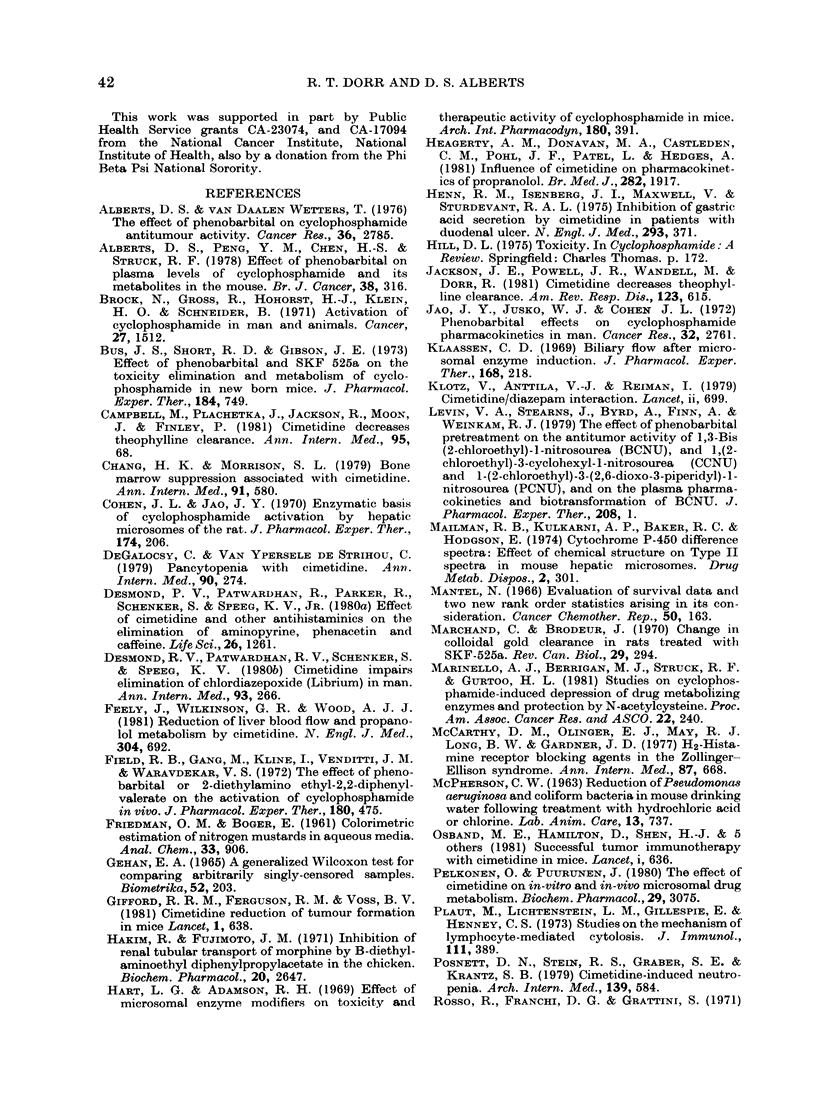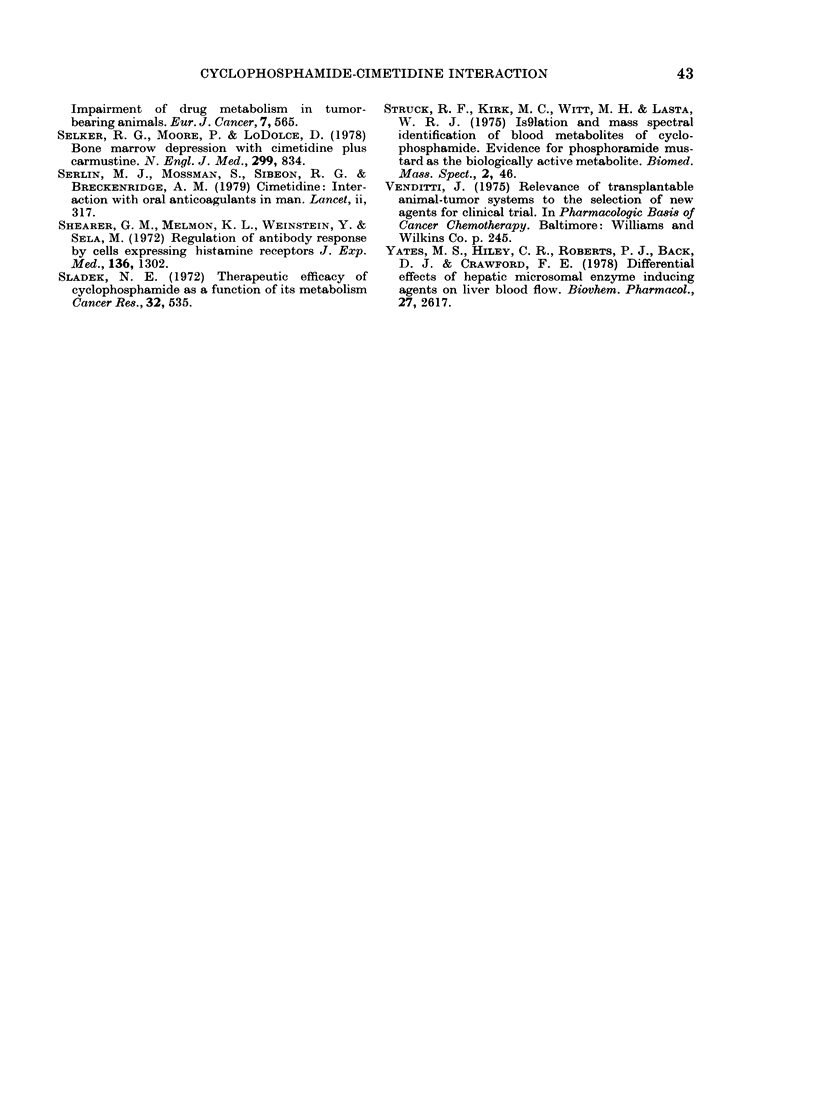# Cimetidine enhancement of cyclophosphamide antitumour activity.

**DOI:** 10.1038/bjc.1982.5

**Published:** 1982-01

**Authors:** R. T. Dorr, D. S. Alberts

## Abstract

Male DBA2 mice were given 10(6) P-388 leukaemic cells i.p. and cimetidine (CMT) at 100 mg/kg 1 day for 10 days, or as a single 100 mg/kg injection 30 min before cyclophosphamide (CTX). CMT significantly prolonged the survival of groups of mice receiving 50, 100 and 200 mg/kg of CTX 3 days after tumour inoculation. Median survival increased by 5.5 days (P less than 0.05), 10 days (P less than 0.05) and 13 days (P less than 0.05) respectively. The addition of CMT had the effect of roughly doubling the CTX dose, without increasing the lethality. CMT produced the only long-term survival seen in the study (1-2/10) CMT alone had no apparent antitumour activity. CMT significantly prolonged mean pentobarbital sleep to 28.6-60 min vs only 10 min for phenobarbital treated mice. Both CMT regimens increased the plasma concentration time products for CTX-induced metabolites (NBP) by about 1.3 fold (in contrast to a 33% reduction with phenobarbital). On average the single-dose CMT regimen produced the greatest effect on survival, on pentobarbital sleep duration and on total NBP reactive species. Probable mechanisms for the CMT-CTX interaction include competitive microsomal enzyme inhibition and/or acutely depressed hepatic blood flow. Caution should be used in combining CMT with full doses of CTX and any other highly metabolized antineoplastic agents in man.


*Br. J. Cancer* (1982) **45**, 35

### CIMETIDINE ENHANCEMENT OF CYCLOPHOSPHAMIDE ANTITUMOUR ACTIVITY

R. T. DORR AND D. S. ALBERTS

*From the Section of Hematology and Oncology, Department of Internal Medicine, and the Cancer Center, College of Medicine, Tucson, Arizona 85724, U.S.A.*

Received 17 August 1981 Accepted 16 September 1981

**Summary.—Male DBA2 mice were given $10^6$ P-388 leukaemic cells i.p. and cimetidine (CMT) at 100 mg/kg 1 day for 10 days, or as a single 100mg/kg injection 30 min before cyclophosphamide (CTX). CMT significantly prolonged the survival of groups of mice receiving 50, 100 and 200 mg/kg of CTX 3 days after tumour inoculation. Median survival increased by 5·5 days ($P<0·05$), 10 days ($P<0·05$) and 13 days ($P<0·05$) respectively. The addition of CMT had the effect of roughly doubling the CTX dose, without increasing the lethality. CMT produced the only long-term survival seen in the study (1–2/10) CMT alone had no apparent antitumour activity. CMT significantly prolonged mean pentobarbital sleep to 28·6–60 min *vs* only 10 min for phenobarbital treated mice. Both CMT regimens increased the plasma concentration time products for CTX-induced metabolites (NBP) by about 1·3 fold (in contrast to a 33% reduction with phenobarbital). On average the single-dose CMT regimen produced the greatest effect on survival, on pentobarbital sleep duration and on total NBP reactive species. Probable mechanisms for the CMT-CTX interaction include competitive microsomal enzyme inhibition and/or acutely depressed hepatic blood flow. Caution should be used in combining CMT with full doses of CTX and any other highly metabolized antineoplastic agents in man.**

CYCLOPHOSPHAMIDE (CTX) is a clinically important anticancer drug which must be metabolized *in vivo* to its alkylating species to show antitumour activity in animals and man (Brock *et al.*, 1971; Sladek, 1972). The initial step of the complex metabolic process involves enzymatic hydroxylation in the 4 position of the oxazophosphorine ring, and is mediated by mixed-function oxygenase; microsomal enzymes concentrated in the mammalian liver. A variety of other drugs used clinically may interact with these enzyme systems and thereby alter the metabolic activation and antitumour effectiveness of CTX (Hart & Adamson, 1969).

Cimetidine (CMT) is a blocker of the $H_2$ histamine receptor and is used in the treatment of ulcer disease (Henn *et al.*, 1975). Recently, CMT has been shown to depress the clearance of a number of microsomally metabolized drugs in rodents (Desmond *et al.*, 1980*a*; Pelkonen & Puurunen, 1980) and in man (Desmond *et al.*, 1980*b*; Klotz *et al.*, 1979; Serlin *et al.*, 1979; Jackson *et al.*, 1981).

Because CMT can alter drug metabolism and is commonly used in cancer patients, we studied the possibility of interaction between CMT and CTX in leukaemic mice. Our results show significant cimetidine-induced enhancement of both the antitumour effects of CTX and the total of alkylating metabolites produced by a single large CTX dose.

### METHODS

*Mice.*—Six to 8-week-old male DBA2 mice weighing ~28 g were used in all experiments. For survival studies mice were divided into treatment groups of 10 and housed 5 per cage on standard wood-chip bedding, and were given acid drinking water and food *ad*

*libitum*. Animals were acclimatized at least 2 weeks before study.

*Tumour*.—P-388 leukaemic cells were collected fresh from the ascitic fluid of 3–4 pretreated mice. The cells were pooled in McCoy's 5A medium (Grand Island Biological Co., Grand Island, N.Y.) and adjusted for i.p. injection to a concentration of $10^6$ cells/mm$^3$. The tumour line was originally obtained from Dr Robert Struck (Southern Research Institute, Birmingham, Ala.) and kept viable by serial ascites transplantations at weekly intervals. The P-388 tumour was selected for this study due to its greater sensitivity to anticancer drugs (Venditti, 1975).

*Drugs and chemicals*.—Cyclophosphamide in parenteral powdered form (Mead Johnson and Co., Evansville, Ind.) was reconstituted in sterile water for injection without preservative, and brought to final concentrations of 5, 10 and 20 mg/ml for i.p. injection. Each dose was given at a constant volume of 0·01 ml/g mouse weight. Cimetidine (CMT) in parenteral solution (Smith, Kline & French, Philadelphia, Pa) was further diluted in preservative free 0·89% NaCl to a final concentration of 25 mg/ml for i.p. injection at a dose of 100 mg/kg/day. Sodium phenobarbital in parenteral solution (Winthrop Laboratories, New York, N.Y.) was added to slightly acidified sterile water up to a concentration of 0·5 mg/ml and exchanged for the routinely used acid drinking water (McPherson, 1963).

Assessment of hepatic microsomal enzyme activity was carried out using sleep duration induced by parenteral sodium pentobarbital (Abbott Laboratories, North Chicago, Ill.) diluted to a concentration of 5 mg/ml in 0·89% preservative free NaCl. Each dose was given i.p. at a volume of 0·01 ml/g mouse weight.

For quantitation of total CTX, the alkylating metabolite, 4-(*p*-nitrobenzyl)-pyridine (NBP) was obtained in powdered form (98% pure) from Aldrich Chemical Co., Milwaukee, Wis. and diluted to a 5% w/v concentration. A standard curve for alkylating activity was constructed using serial dilutions of parenteral mechlorethamine (Merck, Sharp and Dohme, West Point, Pa.). Other chemicals used in the assay were reagent grade.

*Administration schedule of chemotherapeutic agents*.—For assessing survival, 3 dose levels of CTX were used: 50, 100 and 200 mg/kg.

Each dose was given i.p. on the 3rd day after i.p. administration of $10^6$ P-388 cells on Day 1. Two schedules of concomitant CMT administration were evaluated: a 10-day load, 100 mg/kg i.p. Days $-4$ to $+6$ (all 3 CTX dose levels) and a single 100mg/kg i.p. injection 30 min before the CTX (100 and 200 mg/kg only) on Day 3. A phenobarbital-treated group (CTX 200 mg/kg only) was placed on the phenobarbital drinking water on Days $-6$ to $+6$. Control groups ($n=10$ each) included: (1) CTX only at the 3 specified doses i.p. on Day 3, (2) CMT only at 100 mg/kg/day i.p. as a 10-day load and (3) 0·89% saline only administered 0·1 ml i..p and also as a 10-day load (no CTX, no CMT). One treatment group (CTX 200 mg/kg) received both the i.p. CMT and the p.o. phenobarbital as described above.

In 4 other groups of animals ($n=5$ each) microsomal enzyme induction by phenobarbital or CMT was assessed by sleep duration studies following an i.p. pentobarbital dose of 45 mg/kg. Treatment groups included control, phenobarbital loaded, cimetidine loaded, and single-dose cimetidine treated animals.

Survival was assessed by observing for death at 12 h intervals. Survival was statistically analysed by computer, primarily using a generalized Wilcoxon test (Gehan, 1965). In some instances the logrank test was added for comparison of survival patterns, since the Wilcoxon test censors data in favour of early survival differences, whereas the logrank method is weighted in favour of detecting differences in long-term survival (Mantel, 1966).

*Cyclophosphamide alkylating metabolites by NBP Assay*.—Four groups of animals ($n=40$ each) were used to study the production of alkylating metabolites of CTX after i.p. injection of 200 mg/kg. This high dose was historical convention and was necessitated by the sensitivity limits of the assay. This dose is close to the $LD_{10}$ (in BDF1 mice) of ~250–300 mg/kg (Hill, 1975). At serial times after CTX, groups of 4 mice were lightly anaesthetized with ether and the total available blood collected by cardiac puncture into iced heparinized centrifuge tubes. The pooled plasma (~1·5 ml) was separated by centrifugation at 2000 rev/min for 10 min, and stored frozen at $-20$°C for analysis later on the day of collection.

The NBP assay was a modification of the colorimetric method of Friedman & Boger (1961) as described by Alberts & van Daalen

FIG. 1.—Survival curves for groups of mice given 3 doses of cyclophosphamide (CTX): 50 (a), 100 (b) and 200 mg/kg (c) on Day 3 (●) compared to the same schedules plus a 10-day load of cimetidine (CMT) 100 mg/kg i.p. Days −4 to +6 (▲). $10^6$ P-388 leukaemia cells are given i.p. on Day 0. Statistical analysis by Wilcoxon method, gives $P$ for differences as follows: a, 0·045; b, 0·005; c, 0·02 logrank tests for C gives $P = 0·05$.

Wetters (1976). Absorbances were read on a Gilford microflow cell spectrophotometer set at 540 nm. Readings were taken 80 s after production of the blue alkylator chromophore, as specified in the modified assay. The need to use 1ml plasma samples (pooled from 4 animals) did not allow duplicate analysis of individual samples. The levels were then used to generate plasma-time decay curves for NBP. The areas under these decay curves (AUC) were calculated using the trapezoidal rule.

### RESULTS

The addition of CMT to all 3 CTX doses (50, 100 and 200 mg/kg) significantly prolonged the survival of the leukaemic mice, (Fig. 1 and Table I). Median survival increased by 5·5 days (($P < 0·005$) 7·0 days ($P < 0·05$) and by 13·0 days ($P < 0·05$) for the 3 CTX doses respectively. There was no apparent antitumour effect for CMT alone at 100 mg/kg i.p. for 10 days. Survival was identical to a saline-treated group (median survival 13 days). Long-term survival was produced only by the addition of CMT, and an advantage was seen for the single pre-CTX 100 mg/kg CMT administration. This was significantly better than 10 days of CMT treatment at this CTX dose level ($P = 0·0001$, Fig. 2).

At the highest CTX dose (200 mg/kg i.p.) early treatment deaths cancelled CMT effects on median survival. None the less, long-term survival was still produced only in groups receiving concomitant CMT: 10% long-term survival in the single-dose CMT group and 20% long-term survival in the 10-day CMT group. The addition of oral phenobarbital at this high CTX dose did not alter survival. The further addition of CMT to phenobarbital and CTX tended to increase median survival (from 18 days to 28 days) but this difference was not statistically significant.

Thus the most effective treatments in this study were 100 mg/kg CTX plus the single CMT injection (better survival than with 200 mg/kg CTX alone; $P < 0·02$ Wilcoxon, $< 0·05$ logrank Fig. 3) and the 200 mg/kg CTX plus either single-dose or 10-day CMT; the latter producing the highest long term survival rate (Fig. 1c). (The logrank $P$-values were consistently higher than the Wilcoxon values.) This confirms that early survival was most

TABLE I.—*Antitumour survival in DBA leukaemic mice* ($10^6$ *P-388 leukaemia cells on Day* 0)

| CTX dose (mg/kg) | Other treatment | CMT dose‡ | Long-term survivors (10 treated) | Median survival† (days) |
|---|---|---|---|---|
| — | | | 0 | 13 |
| — | Control | 10 | 0 | 13 |
| 50 | Control | | 0 | 19·5 |
| 50 | | 10 | 0 | 25* |
| 100 | Control | | 0 | 24 |
| 100 | | 10 | 0 | 31** } ** |
| 100 | | 1 | 0 | 35** } |
| 200 | Control | | 0 | 18 |
| 200 | Phenobarb § | | 0 | 20 |
| 200 | Phenobarb | 10 | 0 | 28 |
| 200 | | 1 | 0 | 29*** |
| 200 | | 10 | 2 | 33 |

† Survival differences are compared to the appropriate control group receiving CTX only (unless otherwise indicated). For all other comparisons (no symbol) $P \gg 0{\cdot}1$.
*$P<0{\cdot}05$, ** $P<0{\cdot}005$, *** $0{\cdot}05<P<0{\cdot}1$.
‡ 10 = 100 mg/kg/day for 10 days.
§ 0·5 mg/ml in drinking water
1 = single dose of 100/mg/kg.]

FIG. 2.—A comparison of the antileukaemic advantage (in terms of survival) of adding a single CMT dose (100 mg/kg) i.p. 30 min before 100 mg/kg CTX (▲) to the same CTX dose given alone (●). $P<0{\cdot}0001$.

FIG. 3.—A comparison of the survival advantage of combining 100 mg/kg CTX with a single 100 mg/kg CMT injection (▲) over double the CTX dose (200 mg/kg) without CMT (●).

effected by CMT. This also demonstrates that CMT does not enhance CTX lethality, since at 200 mg/kg of CTX, CMT actually prevented early treatment deaths (those occurring before Day 13, the median survival day in untreated control animals).

The same pattern of CMT augmentation was evident in the production of CTX alkylating moieties as detected by the NBP assay (Table II). Most alkylating

TABLE II.—*Areas under the NBP aklylating-activity curve.*

| Groups (of 4 mice) | $HN_2$ equivalents, integrated ($\mu$g/ml min) |
|---|---|
| CTX 200 mg/kg i.p. | 5985 |
| CTX 200/kg i.p. + CMT 10-day | 7560 |
| CTX 200/kg i.p. + Phenobarb | 4090 |
| CTX 200/kg i.p. + CMT × 1 (30 min before CTX) | 8075 |

FIG. 4.—A concentration *versus* time plot of CTX-produced NBP alkylating activity for CTX (200 mg/kg, ●——● and for the same schedule with 100 mg/kg CMT given i.p. as a 10-day load (○– – ○).

FIG. 5.—A concentration *versus* time plot of CTX-produced NBP alkylating activity in phenobarbital-pretreated mice receiving 200 mg CTX i.p. (▲——▲) and those receiving the same CTX dose combined with a single injection of CMT 30 min earlier (■– – ■). Note the delay to peak activity for CMT-treated animals compared with the rapid peak produced by phenobarbital pretreatment. Activity in CMT-treated mice remains substantially higher thereafter than those with CTX alone (●——●, Fig. 4) and especially higher than in phenobarbital-pretreated mice (Fig. 5).

TABLE III.—*Pentobarbital sleep times*

| Group | Sleep time (min ± s.d.) |
|---|---|
| Normal mice | 28·6 ± 4·1 |
| Phenobarbital load | 10·5 ± 3·9 |
| CMT (10-day load) | 62·2 ± 35·9 |
| CMT (single dose, 10 min before) | 111·8 ± 23·5 |

activity was seen in the groups receiving concomitant CMT. In contrast, the addition of phenobarbital substantially reduced the area under the NBP curve to about 2/3 the control quantities. Figs 4 and 5 show the decay curves for NBP in the 4 groups. Phenobarbital caused early high peak NBP activity which diminished rapidly. Both CMT-pretreated groups showed prolongation of CTX-associated NBP activity.

CMT also produced major changes in pentobarbital induced sleep (Table III). The single i.p. CMT injection (10 min before pentobarbital) was most potent, increasing the average sleep time almost 4-fold. Animals loaded 10 days on i.p. CMT (last treated 4 h before pentobarbital) had sleep times increased about 2-fold. The variability in each instance however was relatively large. Phenobarbital-pretreated animals showed substantial enzyme induction, sleeping only one-third as long as the control animals.

### DISCUSSION

Cimetidine, an $H_2$ histamine blocker, is commonly used in the acute and chronic management of gastric and duodenal ulcer, as well as in other pathological hypersecretory conditions [*e.g.* the Zollinger Ellison syndrome (McCarthy *et al.*, 1977)]. In addition to the drug interaction seen in this study, CMT can cause a number of uncommon suppressive haematological reactions, including agranulocytosis (Chang & Morrison, 1979; Posnett *et al.*, 1979) and pancytopenia (DeGalocsy & Van Ypersele de Strihou, 1979); as well as consistent immuno stimulation. The latter mechanism involves augmented lymphocyte blastogenesis (Gifford *et al.*,

1981) and CMT blockade of suppressor T-cell activation, normally mediated through $H_2$ histamine receptors. CMT suppresses both antibody formation (Shearer *et al.*, 1972) and T-mediated cytolysis (Plaut *et al.*, 1973). Recently, CMT has been shown to prevent the metastatic spread of 3LL adenocarcinoma and to extend survival in mice (Osband *et al.*, 1981). Inactivation of suppressor cells by CMT indicated a direct immunological tumoricidal role for the drug. Simultaneously Gifford *et al.* (1981) showed that CMT significantly reduces tumour formation and increases the survival of mice given the EL4 ascites lymphoma or the Mc43 fibrosarcoma. No direct *in vitro* cytoxicity of CMT was seen. This is consistent with our conclusion that CMT alone had no antitumour activity.

The most effective immunostimulative CMT dose in Gifford's study (100 mg/kg/day orally) is identical to the i.p. dose used in the present report. While this dose is much larger than CMT doses clinically recommended in man (up to 20 mg/kg/day), peak murine plasma levels from this dose are comparable to levels obtained in man following standard doses (Gifford *et al.*, 1981). Thus, the CMT dose used in the current study should be of biological significance in man.

In addition to the direct haemotological and immunological effects of CMT, the drug also depresses the clearance of a variety of other drugs whose elimination depends on microsomal metabolism. The list of drugs so effected includes the sedatives chlordiazepoxide (Desmond *et al.*, 1980*b*) and diazepam (Klotz *et al.*, 1979) the oral anticoagulant warfarin (Serlin *et al.*, 1979) the methylxanthines caffeine (Desmond *et al.*, 1980*a*) and theophylline, (Campbell *et al.*, 1981; Jackson *et al.*, 1981) as well as the hepatic-clearance indicator drugs, antipyrine (Klotz *et al.*, 1979) and aminopyrine (Desmond *et al.*, 1980*a*). Furthermore, Pelkonen & Puurunen (1980) found that CMT pretreatment in rats caused significant prolongation of hexobarbital sleep and inhibition of aminopyrine N-demethylation without altering benzo(a)pyrene hydroxylation. In addition pretreatment did not induce hepatic drug metabolism. This pattern is consistent with non-specific CMT inhibition of the cytochrome microsomal enzyme systems including the P-448 and P-450 fractions. Spectral analysis indicates a Type II inhibitory pattern for CMT (believed to be a general property of compounds with a "sterically-accessible" nitrogen atom including imidazoles like CMT [Mailman *et al.*, 1974]).

Cyclophosphamide is an antitumour drug which exerts its cytoxic effects via the generation of alkylating metabolites, principally phosphoramide mustard (Struck *et al.*, 1975). Activation of the drug is initiated by 4-hydroxylation, process predominantly due to hepatic microsomal mixed-function oxidases (Cohen & Jao, 1970). Phenobarbital, a known inducer of diverse hepatic microsomal activities (including P-450), has demonstrated no significant CTX interactions in man (Jao *et al.*, 1972). Yet in experimental animals in this study and others (Alberts *et al.*, 1976; 1978) the addition of this enzyme inducer markedly reduces the total amount of alkylating metabolites from CTX, and its antitumour effects. It should be borne in mind, however, that phenobarbital administration also increases hepatic size and blood flow (Yates *et al.*, 1978) and bile flow (Klaassen, 1969).

Conversely, experimental microsomal enzyme inhibitors such as SKF-525a (diethylaminoethyldiphenyl valerate) depress hepatic blood flow (Hakim & Fujimoto, 1971) and can reverse phenobarbital induction and restore CTX antitumour activity (Alberts *et al.*, 1976). When used alone, SKF-525a reduced CTX lethality in animals without altering its antitumour effectiveness (Hart & Adamson, 1969). This inhibitor can also prolong the half-life of CTX in mice (Bus *et al.*, 1973) an effect identical to the CMT-CTX interaction in the present study. CMT also similarly reduced CTX

lethality in this study while substantially enhancing both early and late tumour survival.

Thus we have demonstrated consistent CMT-induced enhancement of the antitumour effect of CTX in mice. CMT also significantly prolonged phenobarbital sleep, as in the findings of Pelkonen & Puurunen (1980). We were not able to detect a direct antitumour effect of CMT in the P-388 leukaemia system. On average the addition of CMT as a 10-day i.p. load had the survival prolongation effect of roughly doubling the CTX dose (Table I). Also CMT inclusion produced the only long-term survival. In this respect the 10-day CMT load did not dramatically increase survival over a single CMT injection 30 min before CTX. Similarly a single CMT injection produced the largest amount of NBP alkylating species (Table II), and the greatest prolongation of pentobarbital sleep. A significant CMT effect on liver size or total microsomal enzyme mass is not consistent with these results. At least two other reasonable explanations remain for the augmented CTX effects seen in this study: (1) a competitive alteration or blockade of substrate (CTX) sites on hepatic P-450 microsomal enzymes and (2) a significant CMT-induced reduction in liver blood flow, slowing the hepatic extraction of CTX A reduction in liver blood flow has been described for the other classic enzyme inhibitor SKF-525a in animals (Marchand & Brodeur, 1970) and in man; CMT has been shown to depress liver blood flow by up to 25% after acute administration and by 33% following chronic oral administration (Feely *et al.*, 1981).

The results of the NBP-CTX assays are difficult to assess since the assay is not specific for cytotoxic CTX metabolites. Thus inactive as well as active metabolites and parent drug will be quantified. None the less, our analysis demonstrated prolonged retention of NBP-reactive species in CMT-treated mice (Figs 4 & 5). In addition phenobarbital depressed the formation of total NBP-reactive species by about one-third (Table II). This is consistent with pharmacokinetic results using a more specific NPB-reactive, thin-layer chromatography in which a 27% reduction in the AUC for phosphoramide mustard was obtained following the combination of CTX with phenobarbital (Alberts *et al.*, 1978). The more rapid CTX decay in the presence of phenobarbital had also been described earlier (Field *et al.*, 1972; Bus *et al.*, 1973). It is possible that CMT might have even greater effects on orally administered CTX, for which hepatic extraction is significant following absorption. In this regard CMT has recently been shown to substantially increase the bioavailability of oral propranolol, another highly extracted drug (Heagerty *et al.*, 1981).

The metabolism of a number of other clinically used anticancer drugs is mediated to some degree by microsomal enzymes. These include procarbazine, dacarbazine, doxorubicin, hexamethylmelamine and the nitrosoureas. For one such compound, the nitrosourea carmustine (BCNU) a similar pattern to CTX is emerging: phenobarbital pretreatment in rats destroyed the antitimour activity of BCNU (Levin *et al.*, 1979) while concurrent CMT markedly enhanced BCNU-induced marrow depression in a single patient (Selker *et al.*, 1978). It should also be borne in mind, however, that drug metabolism and microsomal enzyme activity may be inhibited by the presence of tumour (Rosso *et al.*, 1971) or by the antitumour drug itself (*e.g.* CTX in Marinello *et al.*, 1981).

In conclusion we have demonstrated that CMT induces (1) statistically significant augmentation of CTX antitumour effects in leukaemic mice and (2) substantial increases in NBP alkylating species following a large CTX dose. It is possible that similar CMT-induced CTX augmentation may be seen in man. Thus, until more definitive studies are completed, caution should be exercised whenever cimetidine is combined with full doses of CTX or any other microsomally metabolized antineoplastic drug.

This work was supported in part by Public Health Service grants CA-23074, and CA-17094 from the National Cancer Institute, National Institute of Health, also by a donation from the Phi Beta Psi National Sorority.

### REFERENCES

ALBERTS, D. S. & VAN DAALEN WETTERS, T. (1976) The effect of phenobarbital on cyclophosphamide antitumour activity. *Cancer Res.*, **36**, 2785.

ALBERTS, D. S., PENG, Y. M., CHEN, H.-S. & STRUCK, R. F. (1978) Effect of phenobarbital on plasma levels of cyclophosphamide and its metabolites in the mouse. *Br. J. Cancer*, **38**, 316.

BROCK, N., GROSS, R., HOHORST, H.-J., KLEIN, H. O. & SCHNEIDER, B. (1971) Activation of cyclophosphamide in man and animals. *Cancer*, **27**, 1512.

BUS, J. S., SHORT, R. D. & GIBSON, J. E. (1973) Effect of phenobarbital and SKF 525a on the toxicity elimination and metabolism of cyclophosphamide in new born mice. *J. Pharmacol. Exper. Ther.*, **184**, 749.

CAMPBELL, M., PLACHETKA, J., JACKSON, R., MOON, J. & FINLEY, P. (1981) Cimetidine decreases theophylline clearance. *Ann. Intern. Med.*, **95**, 68.

CHANG, H. K. & MORRISON, S. L. (1979) Bone marrow suppression associated with cimetidine. *Ann. Intern. Med.*, **91**, 580.

COHEN, J. L. & JAO, J. Y. (1970) Enzymatic basis of cyclophosphamide activation by hepatic microsomes of the rat. *J. Pharmacol. Exper. Ther.*, **174**, 206.

DEGALOCSY, C. & VAN YPERSELE DE STRIHOU, C. (1979) Pancytopenia with cimetidine. *Ann. Intern. Med.*, **90**, 274.

DESMOND, P. V., PATWARDHAN, R., PARKER, R., SCHENKER, S. & SPEEG, K. V., JR. (1980*a*) Effect of cimetidine and other antihistaminics on the elimination of aminopyrine, phenacetin and caffeine. *Life Sci.*, **26**, 1261.

DESMOND, R. V., PATWARDHAN, R. V., SCHENKER, S. & SPEEG, K. V. (1980*b*) Cimetidine impairs elimination of chlordiazepoxide (Librium) in man. *Ann. Intern. Med.*, **93**, 266.

FEELY, J., WILKINSON, G. R. & WOOD, A. J. J. (1981) Reduction of liver blood flow and propanolol metabolism by cimetidine. *N. Engl. J. Med.*, **304**, 692.

FIELD, R. B., GANG, M., KLINE, I., VENDITTI, J. M. & WARAVDEKAR, V. S. (1972) The effect of phenobarbital or 2-diethylamino ethyl-2,2-diphenylvalerate on the activation of cyclophosphamide *in vivo*. *J. Pharmacol. Exper. Ther.*, **180**, 475.

FRIEDMAN, O. M. & BOGER, E. (1961) Colorimetric estimation of nitrogen mustards in aqueous media. *Anal. Chem.*, **33**, 906.

GEHAN, E. A. (1965) A generalized Wilcoxon test for comparing arbitrarily singly-censored samples. *Biometrika*, **52**, 203.

GIFFORD, R. R. M., FERGUSON, R. M. & VOSS, B. V. (1981) Cimetidine reduction of tumour formation in mice *Lancet*, **1**, 638.

HAKIM, R. & FUJIMOTO, J. M. (1971) Inhibition of renal tubular transport of morphine by B-diethylaminoethyl diphenylpropylacetate in the chicken. *Biochem. Pharmacol.*, **20**, 2647.

HART, L. G. & ADAMSON, R. H. (1969) Effect of microsomal enzyme modifiers on toxicity and therapeutic activity of cyclophosphamide in mice. *Arch. Int. Pharmacodyn*, **180**, 391.

HEAGERTY, A. M., DONAVAN, M. A., CASTLEDEN, C. M., POHL, J. F., PATEL, L. & HEDGES, A. (1981) Influence of cimetidine on pharmacokinetics of propranolol. *Br. Med. J.*, **282**, 1917.

HENN, R. M., ISENBERG, J. I., MAXWELL, V. & STURDEVANT, R. A. L. (1975) Inhibition of gastric acid secretion by cimetidine in patients with duodenal ulcer. *N. Engl. J. Med.*, **293**, 371.

HILL, D. L. (1975) Toxicity. In *Cyclophosphamide: A Review*. Springfield: Charles Thomas. p. 172.

JACKSON, J. E., POWELL, J. R., WANDELL, M. & DORR, R. (1981) Cimetidine decreases theophylline clearance. *Am. Rev. Resp. Dis.*, **123**, 615.

JAO, J. Y., JUSKO, W. J. & COHEN J. L. (1972) Phenobarbital effects on cyclophosphamide pharmacokinetics in man. *Cancer Res.*, **32**, 2761.

KLAASSEN, C. D. (1969) Biliary flow after microsomal enzyme induction. *J. Pharmacol. Exper. Ther.*, **168**, 218.

KLOTZ, V., ANTTILA, V.-J. & REIMAN, I. (1979) Cimetidine/diazepam interaction. *Lancet*, ii, 699.

LEVIN, V. A., STEARNS, J., BYRD, A., FINN, A. & WEINKAM, R. J. (1979) The effect of phenobarbital pretreatment on the antitumor activity of 1,3-Bis (2-chloroethyl)-1-nitrosourea (BCNU), and 1,(2-chloroethyl)-3-cyclohexyl-1-nitrosourea (CCNU) and 1-(2-chloroethyl)-3-(2,6-dioxo-3-piperidyl)-1-nitrosourea (PCNU), and on the plasma pharmacokinetics and biotransformation of BCNU. *J. Pharmacol. Exper. Ther.*, **208**, 1.

MAILMAN, R. B., KULKARNI, A. P., BAKER, R. C. & HODGSON, E. (1974) Cytochrome P-450 difference spectra: Effect of chemical structure on Type II spectra in mouse hepatic microsomes. *Drug Metab. Dispos.*, **2**, 301.

MANTEL, N. (1966) Evaluation of survival data and two new rank order statistics arising in its consideration. *Cancer Chemother. Rep.*, **50**, 163.

MARCHAND, C. & BRODEUR, J. (1970) Change in colloidal gold clearance in rats treated with SKF-525a. *Rev. Can. Biol.*, **29**, 294.

MARINELLO, A. J., BERRIGAN, M. J., STRUCK, R. F. & GURTOO, H. L. (1981) Studies on cyclophosphamide-induced depression of drug metabolizing enzymes and protection by N-acetylcysteine. *Proc. Am. Assoc. Cancer Res. and ASCO*. **22**, 240.

MCCARTHY, D. M., OLINGER, E. J., MAY, R. J. LONG, B. W. & GARDNER, J. D. (1977) $H_2$-Histamine receptor blocking agents in the Zollinger–Ellison syndrome. *Ann. Intern. Med.*, **87**, 668.

MCPHERSON, C. W. (1963) Reduction of *Pseudomonas aeruginosa* and coliform bacteria in mouse drinking water following treatment with hydrochloric acid or chlorine. *Lab. Anim. Care*, **13**, 737.

OSBAND, M. E., HAMILTON, D., SHEN, H.-J. & 5 others (1981) Successful tumor immunotherapy with cimetidine in mice. *Lancet*, i, 636.

PELKONEN, O. & PUURUNEN, J. (1980) The effect of cimetidine on *in-vitro* and *in-vivo* microsomal drug metabolism. *Biochem. Pharmacol.*, **29**, 3075.

PLAUT, M., LICHTENSTEIN, L. M., GILLESPIE, E. & HENNEY, C. S. (1973) Studies on the mechanism of lymphocyte-mediated cytolosis. *J. Immunol.*, **111**, 389.

POSNETT, D. N., STEIN, R. S., GRABER, S. E. & KRANTZ, S. B. (1979) Cimetidine-induced neutropenia. *Arch. Intern. Med.*, **139**, 584.

ROSSO, R., FRANCHI, D. G. & GRATTINI, S. (1971)

Impairment of drug metabolism in tumor-bearing animals. *Eur. J. Cancer*, **7**, 565.

SELKER, R. G., MOORE, P. & LODOLCE, D. (1978) Bone marrow depression with cimetidine plus carmustine. *N. Engl. J. Med.*, **299**, 834.

SERLIN, M. J., MOSSMAN, S., SIBEON, R. G. & BRECKENRIDGE, A. M. (1979) Cimetidine: Interaction with oral anticoagulants in man. *Lancet*, ii, 317.

SHEARER, G. M., MELMON, K. L., WEINSTEIN, Y. & SELA, M. (1972) Regulation of antibody response by cells expressing histamine receptors *J. Exp. Med.*, **136**, 1302.

SLADEK, N. E. (1972) Therapeutic efficacy of cyclophosphamide as a function of its metabolism *Cancer Res.*, **32**, 535.

STRUCK, R. F., KIRK, M. C., WITT, M. H. & LASTA, W. R. J. (1975) Is9lation and mass spectral identification of blood metabolites of cyclophosphamide. Evidence for phosphoramide mustard as the biologically active metabolite. *Biomed. Mass. Spect.*, **2**, 46.

VENDITTI, J. (1975) Relevance of transplantable animal-tumor systems to the selection of new agents for clinical trial. In *Pharmacologic Basis of Cancer Chemotherapy*. Baltimore: Williams and Wilkins Co. p. 245.

YATES, M. S., HILEY, C. R., ROBERTS, P. J., BACK, D. J. & CRAWFORD, F. E. (1978) Differential effects of hepatic microsomal enzyme inducing agents on liver blood flow. *Biovhem. Pharmacol.*, **27**, 2617.